# Supplementary material for: Effects of nonantibiotic growth promoter combinations on growth performance, nutrient utilization, digestive enzymes, intestinal morphology, and cecal microflora of broilers
Source: PLoS One. 2023 Mar 30;18(3):e0279950. doi: 10.1371/journal.pone.0279950 (PMC10062635; doi:10.1371/journal.pone.0279950)
Supplement: S1 File — (PDF) [file pone.0279950.s001.pdf]

In vitro digestion test

Animal care and experimental protocols (License no. QAU20210321) were approved by the Animal Care and Use Committee of Qingdao Agricultural University, China. The animals were cared for according to the Animal Care Guidelines of China. Pepsin and 42-d broiler ileal fluid were used for the in vitro digestion test, and the optimal addition levels and combinations of non-antibiotic alternative growth promoters were selected based on dry matter and organic matter digestibility, microbiota of digestive fluid, and antioxidant capacity of digestive fluid. Fig shows the screening process for the in vitro digestion tests.

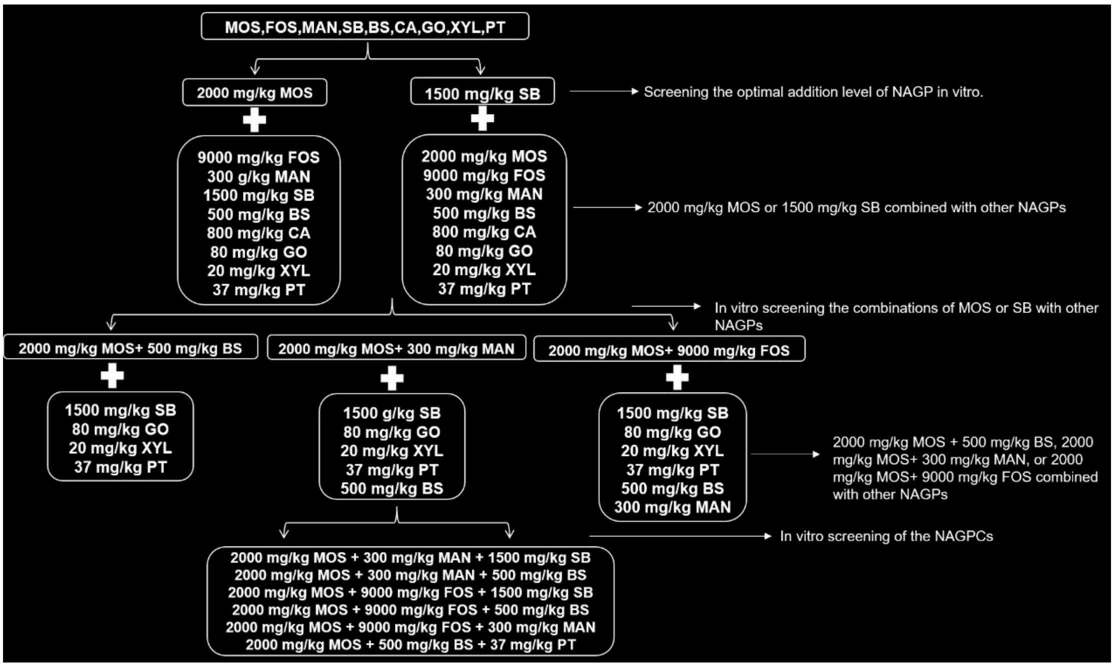

Fig. The screening process of the in vitro digestion test. The biological activities of MAN, PT, XYL, GO, and BS may be affected by pH in the digestive fluid. Because SB and CA can reduce the pH value of digestive fluid and may have synergistic or antagonistic effects with MAN, PT, XYL, GO and BS, a probiotic and an organic acid from MOS, FOS, SB, and CA were selected as substrates to combine with other NAGPs, respectively. NAGP: nonantibiotic alternative growth

promoter; XYL: Xylanase; SB: Sodium butyrate; FOS: Fructose oligosaccharide; PT: Phytase; BS: Bacillus subtilis; MOS: Mannose oligosaccharide; MAN: Mannanase: GO: Glucose oxidase; CA: Guanidinoacetic acid.
